# Identification of LIG1 and LIG3 as prognostic biomarkers in breast cancer

**DOI:** 10.1515/med-2021-0388

**Published:** 2021-11-12

**Authors:** Lin Sun, Xinyu Liu, Siqi Song, Lingjun Feng, Chunying Shi, Zhipeng Sun, Bo Chen, Haiqing Hou

**Affiliations:** Health Management Center, Qilu Hospital (Qingdao), Cheeloo College of Medicine, Shandong University, Qingdao, Shandong, China; Department of Clinical Laboratory, Qilu Hospital (Qingdao), Cheeloo College of Medicine, Shandong University, Qingdao, 266035, China; Department of Thyroid & Breast Surgery, Hospital of Weifang Medical University, Weifang, 261031, China; Department of School of Basic Medicine, College of Medicine, Qingdao University, Qingdao, 266071, China

**Keywords:** LIG, breast cancer, biomarker, prognosis

## Abstract

DNA ligase (LIG) plays a key role in connecting the 3′-OH end of a DNA strand to the 5′-P end of another DNA strand, resulting in the formation of a phosphodiester bond. It has been reported that LIGs (including LIG1, LIG3 and LIG4) play important roles in the occurrence and progression of many cancers. However, the role of LIGs in breast cancer (BC) is still unclear. In this study, we aim to reveal the expression level, function, and prognostic value of LIGs in BC. Bioinformatic tools were used to study the expression level, potential function and prognostic value of LIG1 and LIG3 in BC patients. ENCORI was used to predict microRNAs (miRNAs) that regulate LIG1 and LIG3 and established a valuable miRNA–mRNA regulation network for BC. We found that the expression of LIG1 and LIG3 was upregulated in BC and predicted high relapse-free survival (RFS) in BC patients. Functional annotation analysis was performed to reveal the role of LIG1 and LIG3 in BC. In addition, hsa-miR-22-3p was identified to be potentially involved in the regulation of LIG3. We suggest that LIG1 and LIG3 are novel valuable prognostic biomarkers for BC and has-miRNA-22-3p may be a potential therapeutic target for BC.

## 1 Introduction

Worldwide, the most important cause of women’s premature death is BC [1]. Epidemiological reports released around the world from 1990 to 2017 showed that prevalence and mortality were significantly increased in BC, especially in developing countries and low-income areas. Although mortality has decreased slightly in developed countries, it will continue to increase in the next few years [2,3]. Statistics from the American Cancer Society on female BC showed that the incidence of BC increased slightly by 0.3% annually from 2012 to 2016 [4]. At present, the combination of surgical chemotherapy and radiotherapy is the most commonly used strategy for the therapy of BC patients. In addition, the individualized treatment strategy is also under exploration.

The LIG protein family is found to be the main component of DNA ligases. It participates in some processes, such as DNA replication, and also interacts with multiple components in the body contributing to the DNA damage repair [5,6,7]. LIGs are involved in a number of physiological processes in cells. LIG1 connects Okazaki fragments and participates in nucleotide resection, alkaline resection and repair processes under the interaction of value-added cell nuclear antigens. It also can interact with DNA polymerase-β to participate in other alkaline resection and repair processes. LIG2 is derived from LIG3 through a proteolytic mechanism. LIG3 can be divided into two forms by different splicing methods, including LIG3-α and LIG3-β. LIG3-α participates in the repair process of nucleic acid through DNA repair protein named XRCC1, and LIG3-β is found in male germ cells. LIG4 plays an important role in the V(D)J recombination of DNA double-strand break and the connection of non-comorphic ends [5,8].

It has been reported that the expression level of DNA ligase is dysregulated in various cancer cell lines and has been identified as a marker for DNA damage repair in cancer. The potential value of DNA ligase as an anti-cancer target is gradually being recognized [8,9,10]. Although it has been reported that DNA ligase plays important roles in cancer, the systematic role of LIGs in BC is still not clear.

In this study, the bioinformatics analysis of LIGs in BC was performed, including the mRNA expression analysis, protein–protein interaction (PPI) network analysis, functional annotation enrichment analysis, survival analysis and a miRNA-LIG3 regulation network analysis. We observed that LIG1 and LIG3 were overexpressed in BC and predicted a good prognosis in BC patients. Furthermore, has-miRNA-22-3p was identified to be potentially involved in regulating LIG3. Thus, our research indicates that LIG1 and LIG3 may be novel prognostic and predictive biomarkers for BC.

## 2 Materials and methods

### 2.1 Oncomine analysis

Oncomine [11] (https://www.oncomine.org/resource/login.html) was used to analyze the expression of the LIG protein family in various cancers. Red cells indicated that the LIGs genes were significantly upregulated, while the blue cells indicated that the LIGs genes were significantly downregulated (*p* value: 0.01; fold change: 2; and the rank was set to Top 10%).

### 2.2 CCLE analysis

Cancer Cell Line Encyclopedia [12] (CCLE, https://portals.broadinstitute.org/ccle) is a public online database containing data from cancer patients’ cell lines, which can be used for genomic data research. The mRNA expression levels of LIG1 and LIG3 in different cancer cell lines were detected using CCLE.

### 2.3 GEPIA analysis

Gene Expression Profiling Interactive Analysis [13] (GEPIA, http://gepia.cancerpku.cn/) includes analysis of single gene, cancer type and multiple genes. Using GEPIA, we analyzed the expression levels and correlation between LIG1 and LIG3 in BC tissues.

### 2.4 UALCAN analysis

UALCAN [14] (http://ualcan.path.uab.edu/analysis.html) is a useful and valuable web for analyzing various cancer data. We analyzed clinicopathological characteristics of BC patients through eight groups, including patients’ age, race, gender, cancer stages, subtype, TP53 mutation status, menopause status and nodal metastasis status.

### 2.5 Survival analysis

Using the Kaplan–Meier plotter [15] (http://kmplot.com/analysis/), we observed the survival plots for the LIG1 and LIG3 expression related to relapse-free survival based on logrank *p* value and hazard ratio (HR). The *p* value and HR with a 95% confidence interval are shown in the plots.

### 2.6 cBioPortal analysis

The cBioPortal [16] (https://www.cbioportal.org) data are derived from TCGA, ICGC, GEO and other databases. We showed genomic data and alterations of LIG1 and LIG3 based on the cBioPortal database.

### 2.7 Protein–protein interaction network analysis

STRING [17] (https://string-db.org/) database collects and sorts out interaction proteins related to target genes to predict the relationship between proteins. We established PPI networks of LIG1 and LIG3.

### 2.8 Functional annotation enrichment analysis

DAVID [18] (https://david.ncifcrf.gov/) database can help us identify rich biological entries, especially GO terms and conduct functional enrichment analysis of target genes. Functional annotation enrichment analysis predicted potential roles for genes that actively interacted with LIG1 and LIG3.

### 2.9 ENCORI analysis

ENCORI [19] (http://starbase.sysu.edu.cn/) is a platform for researching the data of miRNA, mRNA, ncRNA, ncRNA, etc. In our study, the expression and prognostic value of miRNA-regulated LIG1 and LIG3 in breast cancer were predicted by ENCORI. The searching options used in this research were as follows: CLIP Data, medium stringency (≥2); Degradome Data, with or without data; Pan-Cancer, 1 Cancer type.

### 2.10 Statistical analysis

The mRNA expression level of LIGs between BC and normal samples was detected to reveal the statistical difference by Student’s *t*-tests. Survival curves of various subtypes in BC patients with different expression levels of LIG1 and LIG3 were drafted based on the Log-rank test and hazard ratio (HR) by the Kaplan–Meier plotter. *p* < 0.05 was considered to be statistically significant.

## 3 Results

### 3.1 Transcription level of LIGs in BC

**Figure 1:**
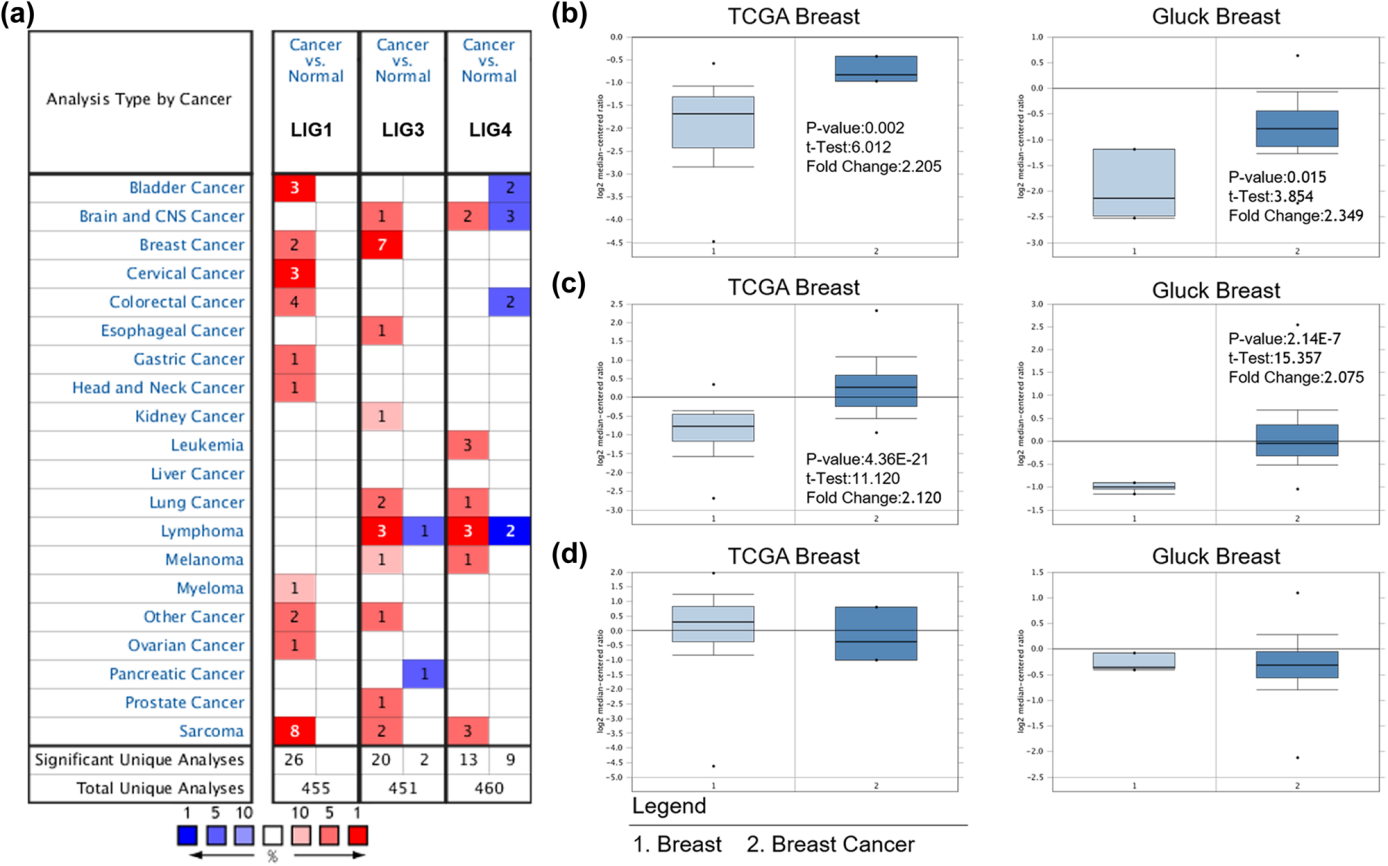
Transcription level of LIGs in different types of cancers. (a) The graph showed the number of relevant datasets that meet the analysis requirements and threshold requirements in each cancer. The transcription level of (b) LIG1 and (c) LIG3 were significantly increased in TCGA and Gluck breast datasets campared with normal samples, but not (d) LIG4.

Currently, the role of LIG family in BC is unclear. In order to identify whether LIGs participated in the occurrence and progression of BC, the Oncomine database was used to analyze the expression of LIGs in BC and normal tissue samples. As shown in Figure 1a, the Oncomine analysis contained a total of 455 unique analyses of LIG1, 451 unique analyses of LIG3 and 460 unique analyses of LIG4. Compared with normal samples, the mRNA expression of LIG1, LIG3 and LIG4 in BC was detected. The expression level of LIG1 was significantly higher in TCGA (*p* value = 0.002, fold change = 2.205) and Gluck breast (*p* value = 0.015, fold change = 2.349) [20] datasets compared with normal samples (Figure 1b). LIG3 was overexpressed in TCGA (*p* value = 4.36 × 10^−21^, fold change = 2.210) and Gluck breast (*p* value = 2.14 × 10^−7^, fold change = 2.075) datasets campared with normal samples (Figure 1c). However, no dataset showed upregulated mRNA expression of LIG4 in BC (Figure 1d).

### 3.2 High expression of LIG1 and LIG3 in BC

**Figure 2:**
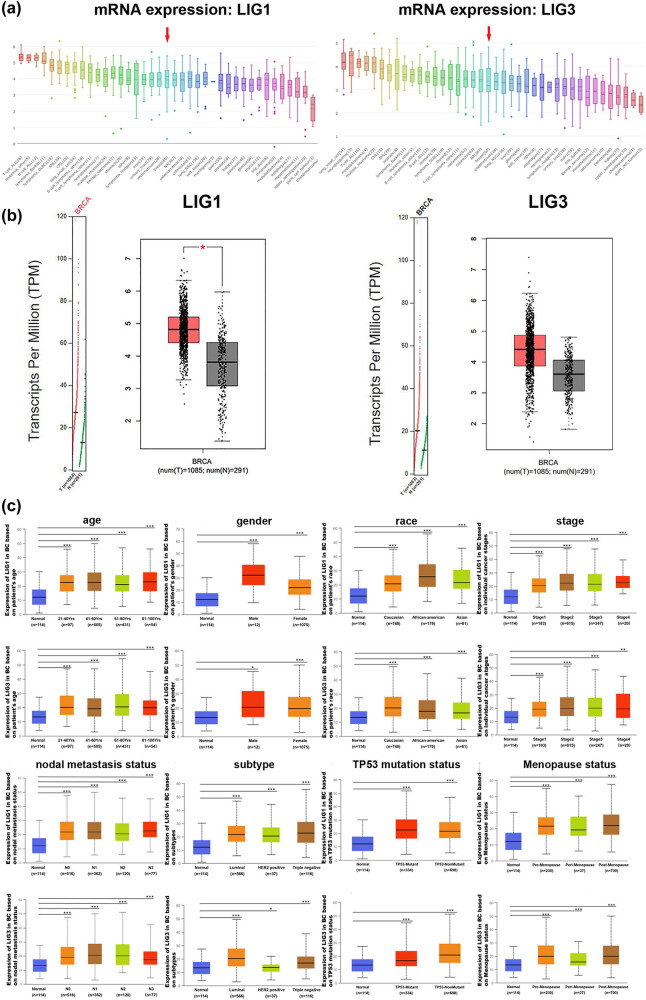
The LIG1 and LIG3 expression levels were upregulated in BC. The expression levels of LIG1 and LIG3 in BC were higher than normal samples based on three aspects, including (a) the mRNA transcription level in BC cell lines (CCLE), (b) the LIG1 and LIG3 expression in BC tissues compared with normal samples (GEPIA), (c) clinicopathological characteristics are shown based on UALCAN (eight groups: age, gender, race, cancer stages, nodal metastasis status, subtype, TP53 mutation status and menopause status). *p < 0.05; **p < 0.01; and ***p < 0.001.

To confirm the upregulation of LIG1 and LIG3 in BC, Cancer Cell Line Encyclopedia (CCLE) analysis was performed to detect the mRNA expression levels of LIG1 and LIG3 in different types of cancer cell lines. As shown in Figure 2a, we found the overexpressed transcription level of LIG1 and LIG3 in BC cell lines. Furthermore, Gene Expression Profiling Interactive Analysis (GEPIA) showed that the mRNA expression level of LIG1 and LIG3 was higher in BC than in normal tissues (Figure 2b). In addition, using the UALCAN database, we analyzed the relationship between the high expression of LIG1 and LIG3 in BC with different clinicopathological characteristics. As shown in Figure 2c, the upregulated expression of LIG1 and LIG3 in BC compared with normal samples significantly related to age, gender, race, cancer stages, nodal metastasis status, subtype, TP53 mutation status and menopause status.

The above results indicate that the upregulated expression of LIG1 and LIG3 may be closely related to the biological characteristics of malignant BC.

### 3.3 Upregulated mRNA expression of LIG1 and LIG3 predicted a good prognosis in patients with BC

**Figure 3:**
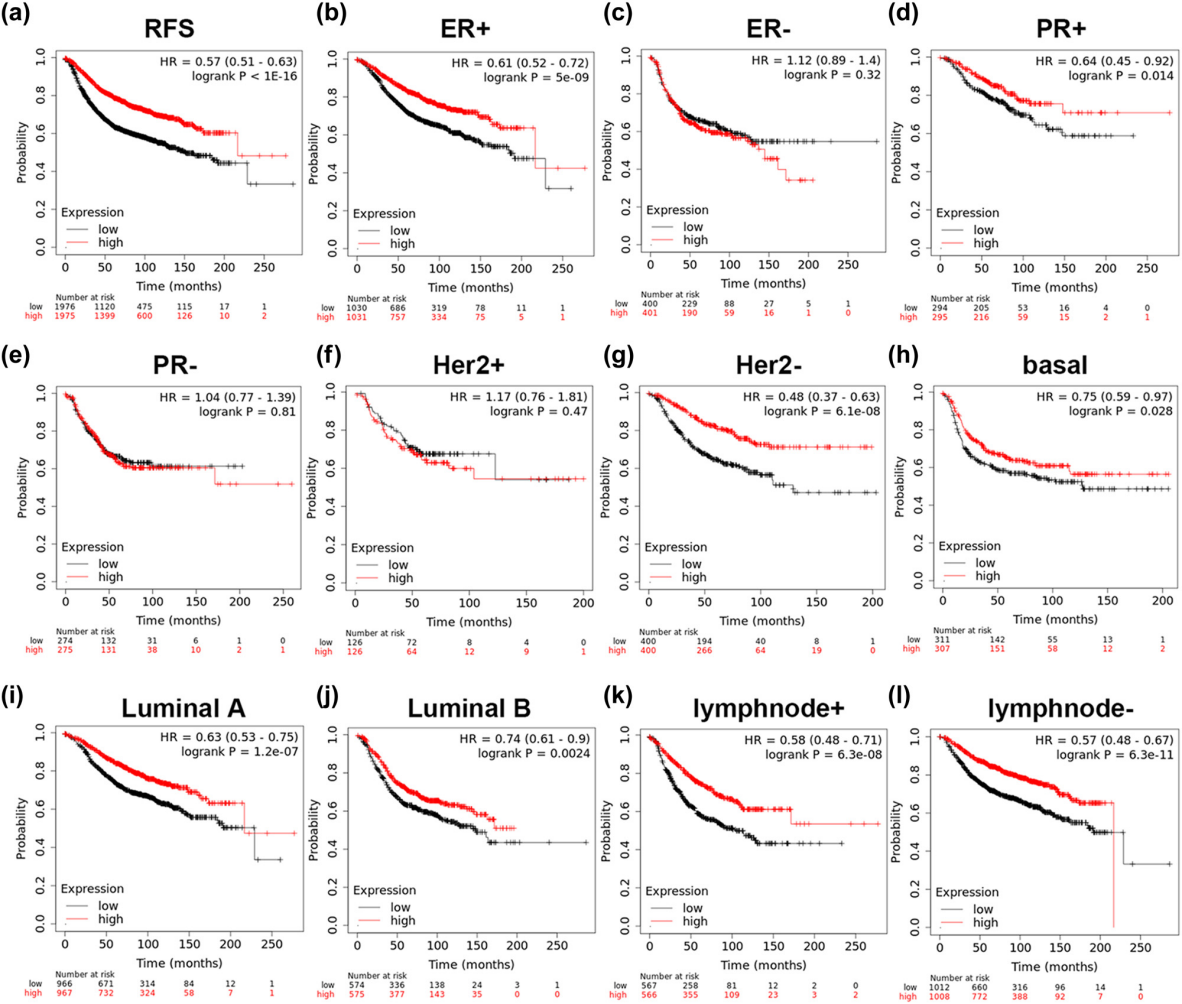
Analysis of the prognostic value of LIG1 in BC. The correlation of LIG1 expression with relapse free survival was analyzed by the Kaplan–Meier plotter in (a) all patients with breast cancer, and various subtypes of BC, including (b) ER-positive, (c) ER-negative, (d) PR-positive, (e) PR-negative, (f) Her2-positive, (g) Her2-negative, (h) basal-like, (i) luminal A, (j) luminal B, and (k) lymphnode-positive and (l) lymphnode-negative.

Using the Kaplan–Meier plotter, the relationship of LIG1 and LIG3 expression with relapse-free survival (RFS) in patients with BC was analyzed. High expression of LIG1 was significantly related to longer RFS of all BC patients (HR = 0.57, *p* < 1 × 10^−16^, Figure 3a). Analysis of different subgroups of BC patients indicated that the high expression of LIG1 was closely related to estrogen receptor-positive (ER-positive) (HR = 0.61, *p* = 5 × 10^−9^, Figure 3b), progesterone receptor-positive (PR-positive) (HR = 0.64, *p* = 0.014, Figure 3d), human epidermal growth factor receptor 2-negative (Her2-negative) (HR = 0.48, *p* = 6.1 × 10^−8^, Figure 3g), basal (HR = 0.75, *p* = 0.028, Figure 3h), luminal A (HR = 0.63, *p* = 1.2 × 10^−7^, Figure 3i), luminal B (HR = 0.74, *p* = 0.0024, Figure 3j), lymph node-positive (HR = 0.58, *p* = 6.3 × 10^−8^, Figure 3k) and lymph node-negative (HR = 0.57, *p* = 6.3 × 10^−11^, Figure 3l). However, the overexpression of LIG1 was not significantly related to some BC subgroups, such as estrogen receptor-negative (ER-negative) (HR = 1.12, *p* = 0.32, Figure 3c), progesterone receptor-negative (PR-negative) (HR = 1.04, *p* = 0.81, Figure 3e) and human epidermal growth factor receptor 2-positive (Her2-positive) (HR = 1.17, *p* = 0.47, Figure 3f).

**Figure 4:**
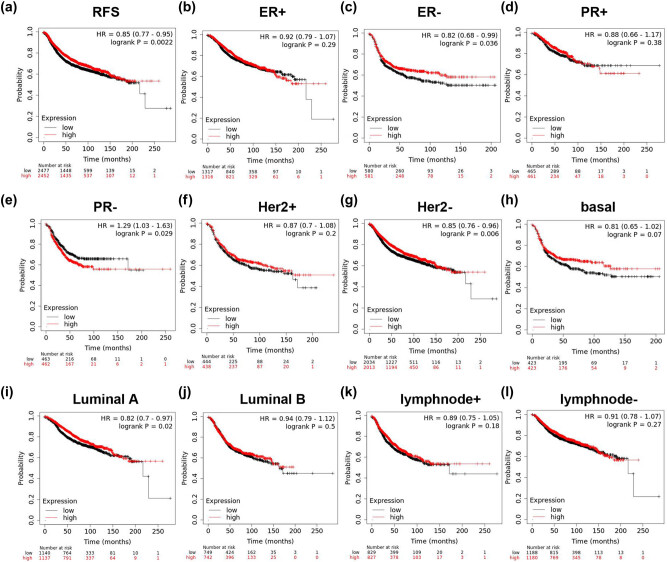
Analysis of the prognostic value of LIG3 in BC. The correlation of LIG3 expression with relapse free survival was analyzed by the Kaplan–Meier plotter in (a) all patients with breast cancer, and various subtypes of BC, including (b) ER-positive, (c) ER-negative, (d) PR-positive, (e) PR-negative, (f) Her2-positive, (g) Her2-negative, (h) basal-like, (i) luminal A, (j) luminal B, (k) lymphnode-positive and (l) lymph node-negative.

The high expression of LIG3 was closely related to longer RFS of all BC patients (HR = 0.84, *p* = 0.0016, Figure 4a) and was also associated with some subgroups of BC, including ER-negative (HR = 0.79, *p* = 0.042, Figure 4c), Her2-negative (HR = 0.85, *p* = 0.006, Figure 4g) and luminal A (HR = 0.82, *p* = 0.02, Figure 4i), but not significantly related to other subgroups, such as ER-positive (HR = 0.92, *p* = 0.29, Figure 4b), PR-positive (HR = 0.88, *p* = 0.38, Figure 4d), Her2-positive (HR = 0.85, *p* = 0.2, Figure 4f), basal (HR = 0.81, *p* = 0.07, Figure 4h), luminal B (HR = 0.94, *p* = 0.5, Figure 4j), lymphnode-negative (HR = 0.89, *p* = 0.18, Figure 4k) and lymphnode-negative (HR = 0.89, *p* = 0.18, Figure 4l). However, high LIG3 expression was related to shorter RFS in PR-negative subtype BC patients (HR = 1.29, *p* = 0.029, Figure 4e).

### 3.4 Alterations of LIG1 and LIG3 in BC patients, protein–protein interaction networks and functional annotation enrichment analysis

Based on the results of the significant high expression level and prognostic value described above, both LIG1 and LIG3 were selected for further analysis.

**Figure 5:**
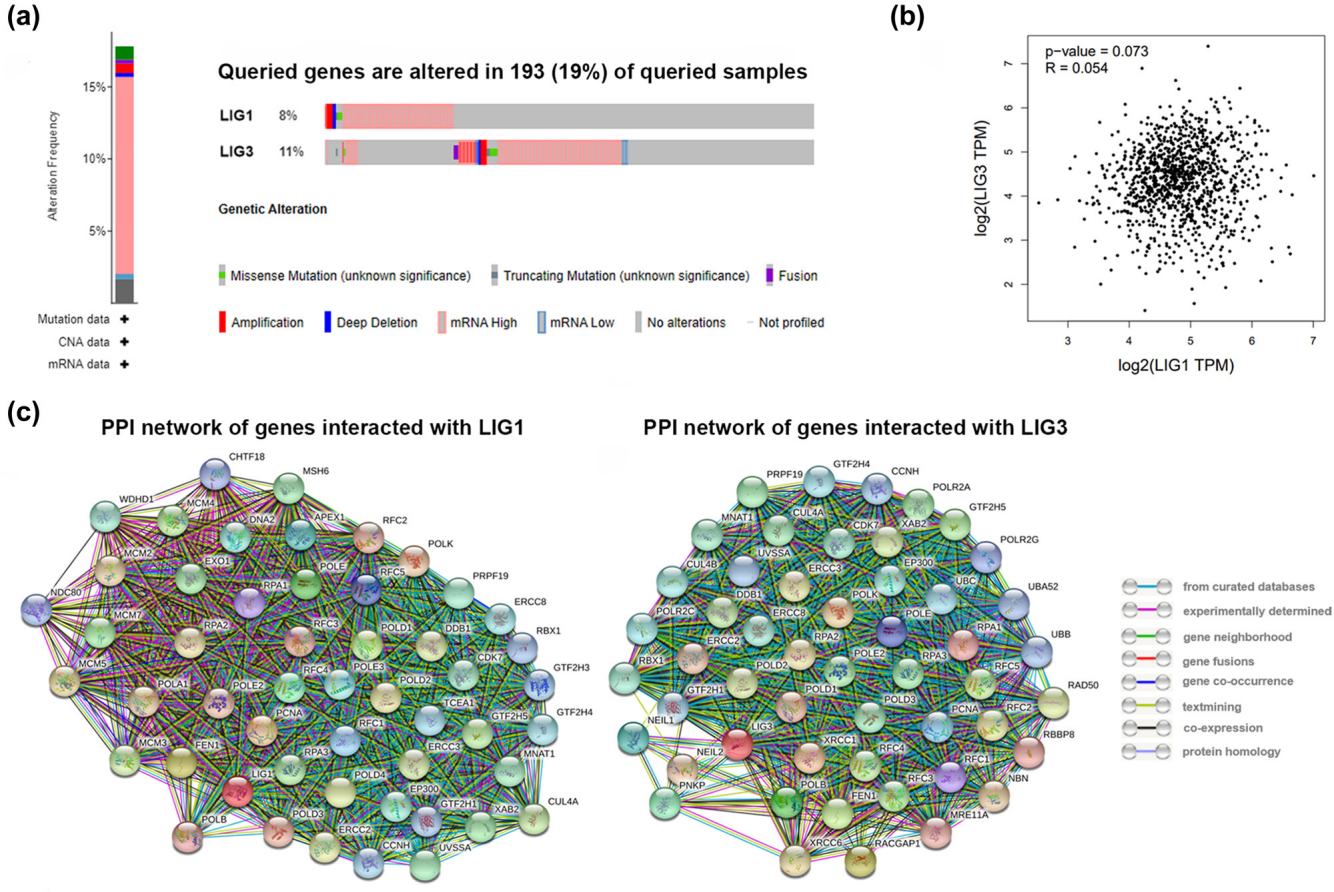
Alterations of LIG1 and LIG3 in BC patients and the protein–protein interaction network. (a) Alterations of LIG1 and LIG3 in BC (cBioportal). (b) No significant correction between LIG1 and LIG3 in BC (GEPIA). (c) Protein–protein interaction networks for LIG1 and LIG3 with the 50 most frequently interacted genes.

Using cBioPortal, a breast-invasive carcinoma dataset was used to analyze LIG1 and LIG3 alterations, including mutations, putative copy-number alterations from GISTIC and mRNA expression *z*-scores relative to diploid samples (RNA Seq V2 RSEM). LIG1 and LIG3 were altered in 198 samples of 1,084 patients with BC (19%) (Figure 5a) and they were mainly high mRNA expression in BC. We also detected the correlation between LIG1 and LIG3 by analyzing their mRNA expressions in BC based on GEPIA database (Pearson’s correction was included). As shown in Figure 5b, there was no significant correlation between LIG1 and LIG3. Next, we constructed protein–protein interaction networks for LIG1 and LIG3 with the 50 most frequently interacted genes (Figure 5c).

Furthermore, the functions of LIG1 and LIG3 and their interaction genes significantly related to LIG1 and LIG3 were predicted by the analysis of gene ontology (GO) based on the Database for Annotation, Visualization and Integrated Discovery (DAVID) database. As shown in Tables 1 and 2, GO enrichment analysis was performed to predict the roles of target host genes based on three aspects, including biological process (BP), cellular component (CC) and (molecular function) MF. LIG1 and LIG3 were associated with some important BPs, such as nucleotide-excision repair and DNA replication.

**Table 1:** Functional annotation enrichment analysis of LIG1 and their interaction genes performed with the DAVID database

	GO term	Count	*p* value	FDR
	GO:0006283 (transcription-coupled nucleotide-excision repair)	33	9.46 × 10^−67^	2.10 × 10^−64^
	GO:0006296 (nucleotide-excision repair, DNA incision, 5′-to lesion)	23	5.78 × 10^−49^	6.41 × 10^−47^
	GO:0033683 (nucleotide-excision repair, DNA incision)	23	1.37 × 10^−48^	1.01 × 10^−46^
	GO:0006260 (DNA replication)	28	4.45 × 10^−43^	2.47 × 10^−41^
BP	GO:0000722 (telomere maintenance via recombination)	19	3.04 × 10^−39^	1.35 × 10^−37^
	GO:0006297 (nucleotide-excision repair, DNA gap filling)	17	1.28 × 10^−36^	4.73 × 10^−35^
	GO:0042769 (DNA damage response, detection of DNA damage)	16	4.65 × 10^−30^	1.47 × 10^−28^
	GO:0006294 (nucleotide-excision repair, preincision complex assembly)	15	3.14 × 10^−29^	8.71 × 10^−28^
	GO:0019985 (translesion synthesis)	14	4.28 × 10^−25^	1.06 × 10^−23^
	GO:0006293 (nucleotide-excision repair, preincision complex stabilization)	12	1.34 × 10^−23^	2.98 × 10^−22^
	GO:0005654 (nucleoplasm)	49	4.26 × 10^−37^	2.86 × 10^−35^
	GO:0005675 (holo TFIIH complex)	8	2.47 × 10^−16^	8.27 × 10^−15^
	GO:0000784 (nuclear chromosome, telomeric region)	13	9.79 × 10^−16^	2.19 × 10^−14^
	GO:0005663 (DNA replication factor C complex)	6	7.58 × 10^−13^	1.27 × 10^−11^
CC	GO:0000439 (core TFIIH complex)	6	1.58 × 10^−11^	2.12 × 10^−10^
	GO:0031390 (Ctf18 RFC-like complex)	5	3.48 × 10^−9^	3.89 × 10^−8^
	GO:0005634 (nucleus)	36	4.66 × 10^−9^	4.46 × 10^−8^
	GO:0042555 (MCM complex)	5	6.25 × 10^−9^	5.24 × 10^−8^
	GO:0005669 (transcription factor TFIID complex)	6	4.48 × 10^−8^	3.33 × 10^−7^
	GO:0043625 (delta DNA polymerase complex)	4	7.76 × 10^−8^	5.20 × 10^−7^
	GO:0003684 (damaged DNA binding)	14	2.30 × 10^−21^	2.44 × 10^−19^
	GO:0003677 (DNA binding)	31	3.71 × 10^−18^	1.97 × 10^−16^
	GO:0003887 (DNA-directed DNA polymerase activity)	10	7.81 × 10^−17^	2.76 × 10^−15^
	GO:0008353 (RNA polymerase II carboxy-terminal domain kinase activity)	8	1.44 × 10^−14^	3.82 × 10^−13^
MF	GO:0008094 (DNA-dependent ATPase activity)	9	3.28 × 10^−14^	6.95 × 10^−13^
	GO:0003689 (DNA clamp loader activity)	6	1.03 × 10^−11^	1.82 × 10^−10^
	GO:0043142 (single-stranded DNA-dependent ATPase activity)	6	4.62 × 10^−11^	7.00 × 10^−10^
	GO:0003678 (DNA helicase activity)	7	6.40 × 10^−11^	8.48 × 10^−10^
	GO:0005515 (protein binding)	47	1.14 × 10^−09^	1.35 × 10^−8^
	GO:0004003 (ATP-dependent DNA helicase activity)	5	2.30 × 10^−6^	2.44 × 10^−5^
	hsa03420:nucleotide excision repair	30	1.37 × 10^−56^	5.20 × 10^−55^
	hsa03030:DNA replication	25	2.33 × 10^−47^	4.43 × 10^−46^
	hsa03430:mismatch repair	16	5.93 × 10^−29^	7.51 × 10^−28^
	hsa03410:base excision repair	12	4.34 × 10^−17^	4.12 × 10^−16^
Path	hsa03022:basal transcription factors	9	3.14 × 10^−10^	2.39 × 10^−9^
ways	hsa03440:homologous recombination	7	2.36 × 10^−8^	1.49 × 10^−7^
	hsa04110:cell cycle	10	7.70 × 10^−8^	4.18 × 10^−7^
	hsa00240:pyrimidine metabolism	8	3.43 × 10^−6^	1.63 × 10^−5^
	hsa05166:HTLV-I infection	10	3.06 × 10^−5^	1.29 × 10^−4^
	hsa00230:purine metabolism	8	1.27 × 10^−4^	4.83 × 10^−4^
	hsa03460:Fanconi anemia pathway	4	0.004878	0.01685056
	hsa05203:viral carcinogenesis	6	0.010385	0.03288545
	hsa04120:ubiquitin-mediated proteolysis	5	0.011929	0.03486920

**Table 2:** Functional annotation enrichment analysis of LIG3 and their interaction genes performed with the DAVID database

	GO term	Count	*p* value	FDR
	GO:0006283∼transcription-coupled nucleotide-excision repair	38	1.05 × 10^−81^	2.77 × 10^−79^
	GO:0006296∼nucleotide-excision repair, DNA incision, 5′-to lesion	25	5.51 × 10^−55^	7.25 × 10^−53^
	GO:0033683∼nucleotide-excision repair, DNA incision	25	1.49 × 10^−54^	1.31 × 10^−52^
	GO:0006297∼nucleotide-excision repair, DNA gap filling	20	5.16 × 10^−46^	3.39 × 10^−44^
BP	GO:0042769∼DNA damage response, detection of DNA damage	20	1.02 × 10^−40^	5.37 × 10^−39^
	GO:0006294∼nucleotide-excision repair, preincision complex assembly	18	1.76 × 10^−37^	7.72 × 10^−36^
	GO:0000722∼telomere maintenance via recombination	16	4.76 × 10^−31^	1.79 × 10^−29^
	GO:0019985∼translesion synthesis	16	4.65 × 10^−30^	1.52 × 10^−28^
	GO:0042276∼error-prone translesion synthesis	14	5.21 × 10^−30^	1.52 × 10^−28^
	GO:0006260∼DNA replication	20	1.03 × 10^−26^	2.71 × 10^−25^
	GO:0005654∼nucleoplasm	50	7.26 × 10^−41^	5.30 × 10^−39^
	GO:0005675∼holo TFIIH complex	7	1.26 × 10^−13^	4.59 × 10^−12^
	GO:0005663∼DNA replication factor C complex	6	6.82 × 10^−13^	1.66 × 10^−11^
	GO:0000784∼nuclear chromosome, telomeric region	9	1.90 × 10^−9^	2.86 × 10^−8^
CC	GO:0005634∼nucleus	36	1.96 × 10^−9^	2.86 × 10^−8^
	GO:0000439∼core TFIIH complex	5	5.75 × 10^−9^	7.00 × 10^−8^
	GO:0031390∼Ctf18 RFC-like complex	4	1.01 × 10^−6^	1.06 × 10^−5^
	GO:0031464∼Cul4A-RING E3 ubiquitin ligase complex	4	2.16 × 10^−6^	1.97 × 10^−5^
	GO:0005662∼DNA replication factor A complex	4	8.12 × 10^−6^	6.59 × 10^−5^
	GO:0043625∼delta DNA polymerase complex	3	4.23 × 10^−5^	3.09 × 10^−4^
	GO:0003684∼damaged DNA binding	19	1.44 × 10^−32^	1.56 × 10^−30^
	GO:0008353∼RNA polymerase II carboxy-terminal domain kinase activity	7	3.41 × 10^−12^	1.28 × 10^−10^
	GO:0008094∼DNA-dependent ATPase activity	8	3.54 × 10^−12^	1.28 × 10^−10^
	GO:0003887∼DNA-directed DNA polymerase activity	7	1.97 × 10^−10^	5.36 × 10^−9^
MF	GO:0003677∼DNA binding	23	2.57 × 10^−10^	5.61 × 10^−9^
	GO:0003689∼DNA clamp loader activity	5	4.35 × 10^−9^	7.90 × 10^−8^
	GO:0005515∼protein binding	45	1.74 × 10^−8^	2.71 × 10^−7^
	GO:0004003∼ATP-dependent DNA helicase activity	6	3.17 × 10^−8^	4.32 × 10^−7^
	GO:0043142∼single-stranded DNA-dependent ATPase activity	4	2.72 × 10^−6^	3.29 × 10^−5^
	GO:0019899∼enzyme binding	8	4.60 × 10^−5^	5.01 × 10^−4^
	hsa03420:nucleotide excision repair	27	2.05 × 10^−48^	6.96 × 10^−47^
	hsa03030:DNA replication	15	9.53 × 10^−23^	1.62 × 10^−21^
	hsa03430:mismatch repair	12	3.19 × 10^−19^	3.61 × 10^−18^
	hsa03410:base excision repair	12	4.34 × 10^−17^	3.69 × 10^−16^
Path	hsa03440:homologous recombination	8	4.41 × 10^−10^	3.00 × 10^−9^
ways	hsa03022:basal transcription factors	8	1.19 × 10^−8^	6.73 × 10^−8^
	hsa00240:pyrimidine metabolism	8	3.43 × 10^−6^	1.66 × 10^−5^
	hsa00230:purine metabolism	8	1.27 × 10^−4^	5.40 × 10^−4^
	hsa05166:HTLV-I infection	8	0.001175	0.00443849
	hsa04120:ubiquitin-mediated proteolysis	6	0.001876	0.00637947
	hsa03450:nonhomologous end-joining	3	0.003118	0.00963706
	hsa03460:Fanconi anemia pathway	4	0.004878	0.01382041
	hsa04110:cell cycle	5	0.008473	0.02216030
	hsa03020:RNA polymerase	3	0.018326	0.04450605

Some pathways significantly associated with the functions of LIG1 and LIG3 alterations in BC were found by KEGG analysis, which were involved in the occurrence and pathogenesis of BC, such as cell cycle, DNA replication, and nucleotide excision repair (Tables 1 and 2).

### 3.5 Regulatory miRNA-regulated LIG3 was identified and the expression and prognostic value of the miRNA in BC patients were analyzed

**Figure 6:**
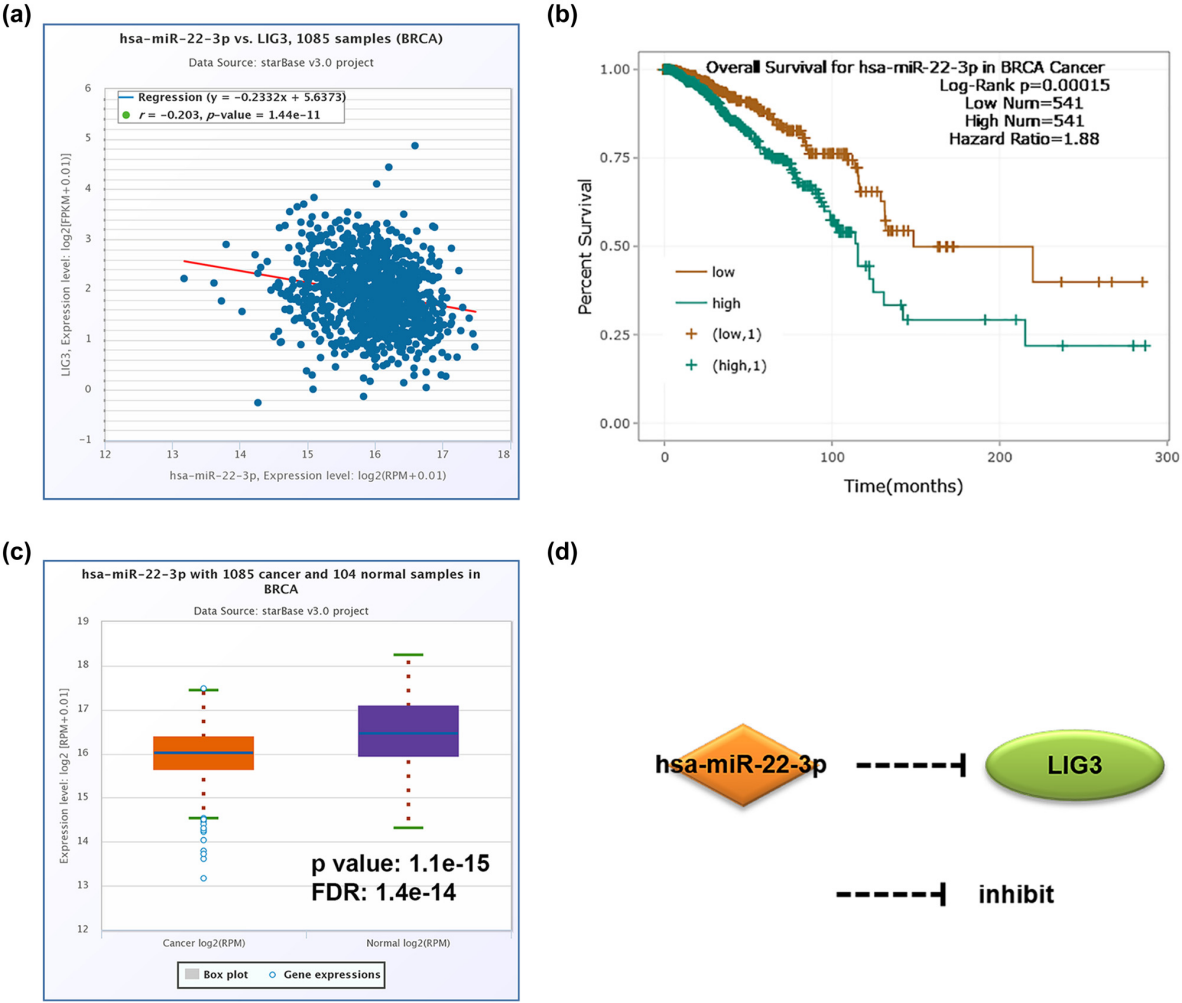
Regulatory miRNA-regulated LIG3 was identified and the expression and prognostic value of the miRNA in BC patients was analyzed. (a) has-miR-22-3p negatively regulated LIG3 expression inBC. (b) The decreased expression of has-miR-22-3p predicted good prognosis for BC patients. (c) The expression of has-miR-22-3p was decreased in BC compared to normal samples. (d) The has-miR-22-3p-LIG3 regulatory network.

A total of eight miRNAs regulating LIG1 and 21 miRNAs regulating LIG3 were predicted based on the ENCORI platform (Tables 3 and 4). Among them, 4 miRNA-LIG1 pairs and 13 miRNA-LIG3 pairs were negatively correlated. However, only one miRNA-LIG3 pair was significantly negatively correlated, and the decreased miRNA expression predicted longer overall survival in BC patients. As shown in Figure 5a–c, we observed that the expression level of hsa-miR-22-3p was decreased in BC compared with normal samples and predicted a good prognosis for BC patients. The above results suggest that the established miRNA-LIG3 regulatory network (Figure 5d) may be a potential prognostic marker of BC and therapeutic target for BC patients (Figure 6).

**Table 3:** Correlation of miRNA-LIG1 pairs identified by the ENCORI database

No.	miRNA	Coefficient-*R*	*p*-value
1	hsa-miR-485-5p	−0.106	4.62 × 10^−4^
2	hsa-miR-4731-5p	−0.065	3.19 × 10^−2^
3	hsa-miR-524-3p	−0.001	9.84 × 10^−1^
4	hsa-miR-676-3p	−0.026	3.97 × 10^−1^
5	hsa-miR-3194-5p	0.028	3.54 × 10^−1^
6	hsa-miR-4761-5p	0.022	4.79 × 10^−1^
7	hsa-miR-525-3p	0.013	6.64 × 10^−1^
8	hsa-miR-3194-5p	0.028	3.54 × 10^−1^

**Table 4:** Correlation of miRNA-LIG3 pairs identified by the ENCORI database

No.	miRNA	Coefficient-*R*	*p*-value
1	hsa-miR-3145-3p	−0.005	8.65 × 10^−1^
2	hsa-miR-381-3p	−0.150	6.61 × 10^−7^
3	hsa-miR-23b-3p	−0.026	3.94 × 10^−1^
4	hsa-miR-187-3p	−0.095	1.81 × 10^−3^
5	hsa-miR-518b	−0.028	3.57 × 10^−1^
6	hsa-miR-518f-3p	−0.092	2.43 × 10^−3^
7	hsa-miR-579-3p	−0.041	1.76 × 10^−1^
8	hsa-miR-340-5p	−0.028	3.48 × 10^−1^
9	hsa-miR-22-3p	−0.203	1.44 × 10^−11^
10	hsa-miR-1179	−0.062	4.21 × 10^−2^
11	hsa-miR-29c-3p	−0.013	6.73 × 10^−1^
12	hsa-miR-29a-3p	−0.152	4.69 × 10^−7^
13	hsa-miR-381-3p	−0.150	6.61 × 10^−7^
14	hsa-miR-185-5p	0.040	1.88 × 10^−1^
15	hsa-miR-455-3p	0.028	3.51 × 10^−1^
16	hsa-miR-29b-3p	0.015	6.29 × 10^−1^
17	hsa-miR-4677-3p	0.016	5.93 × 10^−1^
18	hsa-miR-518a-3p	0.007	8.06 × 10^−1^
19	hsa-miR-330-3p	0.014	6.45 × 10^−1^
20	hsa-miR-664b-3p	0.013	6.58 × 10^−1^
21	hsa-miR-375	0.111	2.34 × 10^−4^

## 4 Discussion

At present, cancer has become the main threat to people’s life and health. According to data released by the American Cancer Society in 2020, 1,806,590 new cancer cases and 606,520 cancer deaths are expected to occur in the United States, and BC is still the most common cause of cancer incidence and death in women [3,21]. To date, more and more suggestions are put forward in clinical applications to design individualized diagnosis and treatment plans for BC patients.

Many BC markers have been found, such as tissue markers, genetic markers and serum markers [22,23,24]. But more valuable biomarkers are still needed to be found to complete the personalized diagnosis and therapy for BC patients. It is worth mentioning that biomedical researchers have established many bioinformatics databases to explore valuable biomarkers for various types of cancer, such as Oncomine, CCLE, GEPIA, UALCAN, Kaplan–Meier plotter, cBioPortal, STRING, DAVID and ENCORI [11,12,13,14,15,16,17,18,19].

DNA damage is considered a hallmark of cancer, and protein mutations that maintain the fidelity of the genome are associated with many cancers. The fracture of double-strand DNA seems to be the most harmful type of DNA damage. The nonhomologous end-joining (NHEJ) pathway is a mechanism for repairing DNA double-strand break. DNA replication may be also regulated by the proteins involved in NHEJ [25]. In recent years, LIGs have been reported to be involved in the development of many cancers, such as lung cancer, nasopharyngeal cancer, rectal adenocarcinoma and BC [25,26,27,28,29,30,31]. To our knowledge, the relationship between the expression of LIG1 and LIG3 and prognostic value for BC patients is still not clear. Therefore, our bioinformatics research explored the LIG1 and LIG3 expression and prognostic value in BC patients, contributing to the further knowledge of LIG1 and LIG3 in BC.

It has been reported that the LIG1 expression in BC is upregulated [32]. However, the LIG3 expression in BC is still not clear. Our study showed that the expression of LIG1 and LIG3 in BC samples was significantly increased, but not LIG4. Furthermore, survival analysis indicated that increased expression of LIG1 and LIG3 was significantly related to longer RFS in all BC patients, indicating that LIG1 and LIG3 had good prognostic value in BC patients. Liao et al. also reported that high LIG3 expression was related to a good prognosis for BC patients [33]. However, the poor prognostic value of LIG3 in PR-negative subtype BC patients needs to be noticed.

In order to explore the role of LIG1 and LIG3 in BC, networks of genes that interacted with LIG1 and LIG3 were, respectively, constructed and GO enrichment analysis was performed to find possible target proteins that interacted with LIG1 and LIG3. We found that LIG1 and LIG3 alterations were related to the nucleotide-excision repair and DNA replication. It also has been reported that LIG1 and LIG3 were related to the nucleotide-excision repair and DNA replication [34,35]. In addition, some pathways significantly associated with the functions of LIG1 and LIG3 alterations in BC were found through KEGG analysis, which were involved in the tumorigenesis and pathogenesis of BC, such as cell cycle, DNA replication, and nucleotide excision repair. It has been reported that cell cycle, DNA replication and nucleotide excision repair were closely related to the occurrence and progression of multiple cancers [36,37,38]. However, the carcinogenic and pathological roles of LIG1 and LIG3 in BC need to be further clarified.

MicroRNAs can be used as potential oncogenes or tumor suppressor genes, which are associated with the progression and treatment of tumors, indicating that they may become potentially valuable diagnosis and prognosis biomarkers [39]. Therefore, we screened miRNAs that may regulate LIG1 and LIG3. We found that hsa-miR-22-3p (negatively correlated with LIG3) had low expression in BC and predicted a good prognosis for BC patients. In previous reports, hsa-miR-22-3p was inhibited by lncRNA DGCR5 to promote the progression of lung adenocarcinoma [40]. However, the role and prognostic value of hsa-miR-22-3p in BC are still unclear. Our research could enrich the role of the expression and prognostic value of hsa-miR-22-3p in BC, which was helpful for the discovery of early BC markers and precise treatment targets.

In conclusion, we confirmed that the expression levels of LIG1 and LIG3 in BC were upregulated and related to a good prognosis for BC patients. Furthermore, the clinicopathological characteristics and related functional annotations of LIG1 and LIG3 were also displayed to deepen their understanding of their role in BC. In addition, we also identified a new miRNA that can negatively regulate LIG3, and revealed the expression level and prognostic significance of hsa-miR-22-3p. Our study suggests that LIG1, LIG3 and hsa-miR-22-3p are involved in the progression of BC and may become potential markers and precise treatment targets.

## Abbreviations

LIG: DNA ligase
BC: breast cancer
GEPIA: gene expression profiling interactive analysis
miRNA: microRNA
GO: gene ontology
BP: biological process
CC: cellular component
MF: molecular function
KEGG: Kyoto encyclopedia of genes and genomes
DAVID: database for annotation, visualization and integrated discovery
PPI: protein–protein interaction
CCLE: cancer cell line encyclopedia
HR: hazard ratio
